# Complete Genome Sequence of *Bradyrhizobium* sp. Strain ORS3257, an Efficient Nitrogen-Fixing Bacterium Isolated from Cowpea in Senegal

**DOI:** 10.1128/MRA.01449-18

**Published:** 2019-01-17

**Authors:** Antoine Le Quéré, Djamel Gully, Albin Teulet, Elisabeth Navarro, Daniel Gargani, Joël Fardoux, Stéphane Cruveiller, Marc Neyra, Eric Giraud, Tatiana Krasova Wade

**Affiliations:** a IRD, Laboratoire Commun de Microbiologie, UR040, ISRA, UCAD, Centre de Recherche de Bel Air, Dakar, Senegal; b IRD, Laboratoire des Symbioses Tropicales et Méditerranéennes, UMR LSTM, Université de Montpellier, CIRAD, Montpellier SupAgro, Montpellier, France; c CIRAD, Biologie et Génétique des Interactions Plante-Parasite, UMR BGPI, Université de Montpellier, INRA, Montpellier SupAgro, Montpellier, France; d CEA, Institut de Biologie François Jacob, Genoscope, Laboratoire d’Analyses Bioinformatiques pour la Génomique et le Métabolisme, UMR-CNRS 8030 Génomique Métabolique, Université d’Évry-Val-d’Essonne, Université Paris-Saclay, Évry, France; Indiana University, Bloomington

## Abstract

Here, we report the complete genome sequence of Bradyrhizobium sp. strain ORS3257, which forms efficient symbioses with cowpea, peanut, or groundnut. These genomic data will be useful to identify genes associated with symbiotic performance and host compatibility on several legumes, including Aeschynomene species, with which a Nod-independent type III secretion system (T3SS)-dependent symbiosis can be established.

## Abstract (precis)

Here, we report the complete genome sequence of Bradyrhizobium sp. strain ORS3257, which forms efficient symbioses with cowpea, peanut, or groundnut.

## ANNOUNCEMENT

Bradyrhizobium sp. strain ORS3257 was isolated from a root nodule of Vigna unguiculata collected in Bambey, Senegal, in 1999 (1). Being an efficient nitrogen-fixing symbiont on various Vigna unguiculata cultivars and a good competitor for nodule occupancy (2, 3), ORS3257 develops efficient symbioses on other tropical legumes of agronomical importance (peanut and groundnut). In addition, this strain can nodulate some Aeschynomene species using an alternative symbiotic process that does not rely on Nod factor synthesis but on a functional type III secretion system (T3SS) (4). Considering its agronomic relevance and its original symbiotic properties, ORS3257, which might be related to the recently described species Bradyrhizobium vignae (5), is an interesting strain to investigate at the genomic level.

In this study, we have obtained the complete genome sequence of Bradyrhizobium sp. strain ORS3257 using the Pacific Biosciences (PacBio) sequencing technology. ORS3257 was grown in liquid yeast malt (YM) medium (6), and genomic DNA was extracted as described by Wilson (7). Libraries were prepared using the Pacific Biosciences 20-kb library preparation protocol. A total of 89,698 polymerase reads with a mean read length of 14,225 bp were generated, which led to a total of 1,277 Mb, with an average coverage of 85-fold. *De novo* assembly of the read sequences was performed using continuous long reads according to the Hierarchical Genome Assembly Process (HGAP) version 3 workflow (DevNet; Pacific Biosciences), as available in the SMRT Analysis software version 2.3.0. Circularization of contigs was performed using the Minimus2 software (Amos package) (8). The sequence was polished sequentially with the RS_Resequencing.1 software (SMRT Analysis version 2.3.0) and Pilon software (version 1.21) (9) using available transcriptomic data (HiSeq 2000; Illumina) that mapped to all predicted coding sequences with a median coverage of 145×. This enabled the correction of 2 nucleotides only, demonstrating the high quality of the assembled genome sequence reported.

The genome of ORS3257 comprises one circular chromosome of 8,156,021 nucleotides, with a GC content of 63.34%. A total of 8,271 coding sequences and 99 RNA genes were predicted using the MicroScope platform (10). A symbiotic island of 730 kb containing *nod*, *nif,* and T3SS genes can be distinguished. Most of the *nod* genes are clustered in a main region comprising the 3 regulator-encoding genes *nodD1*, *nodD2*, and *nolA* in one direction and *nodY*, *nodA*, *nodB*, *nodC*, *nodS*, *nodU*, *nodI*, *nodJ*, and *nodZ* in the opposite direction. The T3SS gene cluster contains all the genes required for the formation of a functional T3SS apparatus (11). Notably, this includes the *nopX* translocon-encoding gene, which is required for injection of effector proteins into host cell and which is not found in several Bradyrhizobium strains, including USDA110. Furthermore, 11 rhizobial T3SS effector homologs were found (*nopC, nopM1, nopM2, nopM3, nopL, nopT, nopP1, nopP2, nopAC, nopAR,* and *nopBW*), all spread within the symbiotic island.

The genomic sequence data reported here will be useful for identifying the effectors governing the establishment of the Nod-independent T3SS-dependent symbiotic process with some Aeschynomene species and the genes that are important for host compatibility and performance of the Bradyrhizobium ORS3257 strain to interact efficiently with several tropical legumes of agronomic importance.

### Data availability.

This genome sequence has been deposited in DDBJ/ENA/GenBank under the accession no. LS398110. The PacBio and HiSeq raw sequence reads used in this study are available from GenBank under the accession no. PRJNA507707 and PRJNA507934, respectively.
